# Placebo-Controlled Trials in the Management of Crohn’s Disease: An Umbrella Review of Meta-Analyses

**DOI:** 10.3390/medsci13010012

**Published:** 2025-01-29

**Authors:** Richard Silva, José Nunes de Azevedo, Jorge Pereira Machado, Jorge Magalhães Rodrigues

**Affiliations:** 1 Clínica Médica Dr. Richard, 3700-317 São João da Madeira, Portugal; 2 TECSAM, Tecnologia e Serviços Médicos SA, 5370-530 Mirandela, Portugal; 3 ICBAS, School of Medicine and Biomedical Sciences, University of Porto, 4050-313 Porto, Portugal; 4 CBSin—Center of BioSciences in Integrative Health, 4000-105 Porto, Portugal

**Keywords:** Crohn, Crohn’s disease, inflammatory bowel diseases, management, drugs, medication, treatment

## Abstract

Introduction: Crohn’s disease is a chronic inflammatory bowel disease characterized by abdominal pain, diarrhea, and other symptoms. It can lead to significant complications and impact patients’ quality of life. Therefore, effective management strategies are essential for improving outcomes. Methods: To assess the efficacy of the treatments for Crohn’s disease, this umbrella review systematically addresses systematic reviews and meta-analyses on Crohn’s disease management published between 2013 and 2023. The quality of the included studies was assessed using the National Institutes of Health’s quality assessment tool. Results: Sixteen studies were included, evaluating various interventions for the induction and maintenance of remission. These included biologic agents (anti-TNF agents, anti-IL-12/23p40 antibodies, and integrin receptor antagonists), antimetabolites, and corticosteroids. Conclusions: The findings suggest that biologic agents may be promising options for both the induction and maintenance of remission in Crohn’s disease. Antimetabolites and corticosteroids may be effective in certain cases, but their efficacy and safety profiles require further investigation. The included studies varied in quality and sample size. More research is needed to confirm the findings and establish optimal treatment strategies. Moreover, while biologic agents show promise, the optimal management of Crohn’s disease requires further research. A personalized approach considering patient factors and disease characteristics is crucial for optimizing outcomes.

## 1. Introduction

Crohn’s disease is an inflammatory bowel disease characterized by an irregular and sporadic inflammatory pattern that extends through the entire thickness of the affected tissue wall [1]. Predominantly, the regions most frequently impacted include the small intestine, particularly the terminal ileum, as well as the colon [2]. Crohn’s disease is linked to a multitude of pathophysiological complications, and its clinical symptoms exhibit variability based on the specific location of the disease within the body [3,4]. Usually, it manifests with abdominal pain, fever, and noticeable clinical indicators of bowel obstruction. It can also lead to diarrhea with the passage of blood, mucus, or a combination of both [3].

Individuals with Crohn’s disease often undergo frequent hospitalizations, numerous surgical procedures, and grapple with compromised quality of life as a result of complications arising from the disease [1]. Such complications vary from stenosis or fistulas to early small bowel and colorectal cancer [2,3,5,6] but may also manifest extraintestinally as arthritis, aphthous stomatitis, uveitis, erythema nodosum, ankylosing spondylitis, psoriasis, pyoderma gangrenosum, or primary sclerosing cholangitis [7].

Beyond its evident health implications, Crohn’s disease can exert a significant influence on various aspects of patient’s lives, encompassing their educational pursuits, occupational endeavours, as well as their social and familial interactions [1,8,9], often leading to decreased mental health and mental illness [10,11,12,13,14,15].

Similar to numerous other immune-related disorders, the exact cause of inflammatory bowel diseases like Crohn’s disease remains unclear [16]. Nonetheless, it is widely acknowledged to be a multifaceted condition in which factors such as genetics, environmental influences, immune system irregularities, and disruptions in the microbiome collectively contribute to an aberrant immune reaction. This reaction disrupts the balance of the mucosal environment, impacting mucosal homeostasis [17].

Due to its escalating incidence and prevalence across all ethnic groups, Crohn’s disease is becoming a matter of growing significance for a diverse array of healthcare practitioners, owing to the systemic nature of the disorder [1,3]. In the past fifty years, significant advancements in comprehending the immuno-pathogenesis of Crohn’s disease, coupled with subsequent enhancements in pharmacological interventions, have empowered clinicians to recognize the potential achievable through the modulation of the immune–inflammatory pathway [18]. The perspectives have shifted from mere symptom control to achieving clinical and endoscopic remission. This shift aims not only to diminish the protracted use of corticosteroids but also to forestall the enduring complications and disability associated with the condition [18]. However, caring for patients with Crohn’s disease introduces distinctive challenges since therapy decisions must consider a multitude of unique patient characteristics [3,19]. The disease can be classified based on the severity, location, extent, and likelihood of complications. Additionally, it can be further stratified according to how responsive it is to medical interventions [3,19]. For this reason, a thorough diagnosis is of crucial importance for the correct management of Crohn’s disease and should not be overlooked.

The primary objective of the study is to comprehensively evaluate and synthesize the existing body of systematic reviews and meta-analyses pertaining to the management strategies employed for Crohn’s disease. By conducting an umbrella review, this study aims to consolidate and critically assess the diverse range of interventions, therapeutic approaches, and outcomes documented across multiple systematic reviews. This comprehensive analysis intends to provide a robust and up-to-date overview of the effectiveness, limitations, and gaps in the current landscape of Crohn’s disease management, aiding clinicians, researchers, and policymakers in making informed decisions and shaping future research directions.

## 2. Methodology

### 2.1. Search Strategy

The researchers systematically searched PubMed, the Cochrane Database of Systematic Reviews, Science Direct, the Scientific Electronic Library Online (Scielo), the Latin American and Caribbean Health Sciences Literature (LILACS), and the Journal Storage (JSTOR) from 2013 to 10 August 2023, to find systematic reviews with meta-analyses of interventions in Crohn’s disease.

The search strategy used the following formula: ((Crohn’s disease) OR (granulomatous colitis) OR (Crohn disease) OR (granulomatous enteritis) OR (regional enteritis)) AND ((management) OR (treatment) OR (therap*)) and then filtered the results by systematic review and meta-analysis. Results were added to Rayyan [20] where duplicates were removed, and two researchers blindly screened titles and abstracts.

Conflicts were resolved through dialogue to achieve unanimity in the decision, and a third reviewer was available to settle any disagreements. This method was also applied for the full-text assessment that was performed after full-text retrieval.

The study’s protocol was registered in the International Prospective Register of Systematic Reviews (PROSPERO CRD42023453101).

### 2.2. Eligibility Criteria

Systematic reviews with meta-analyses, incorporating randomized placebo-controlled trials involving human adult subjects and authored in English, Portuguese, Spanish, or French, were included in the study. Additionally, the review only considered studies conducted within the past decade and centred on assessing single therapies on the induction of remission or remission control of Crohn’s disease, therefore excluding treatment combinations or comparisons and studies focusing on post-operative outcomes.

Studies on the pediatric population were also excluded as well as articles addressing overall inflammatory bowel diseases or treatments not recommended in the clinical guidelines of the American Gastroenterological Association [21].

### 2.3. Quality Assessment

We utilized the Quality Assessment Tool for Systematic Reviews and Meta-Analyses developed by the National Institutes of Health [22]. This tool comprises eight distinct inquiries designed to evaluate potential deficiencies in study methods or execution. The studies’ quality is rated as either “good”, “fair”, or “poor”, based on criteria established by the authors. The authors deliberated on the evaluation parameters and determined that articles with either no major flaws or a maximum of 2 flaws would be categorized as “good”, 1 major flaw or a maximum of 3 flaws as “fair”, and more than 1 major flaw or more than 3 flaws as “poor”.

### 2.4. Data Extraction

Two investigators conducted the data extraction process, and in instances of discord, a discussion with a third researcher was performed to achieve consensus. For each eligible article, details such as authorship, publication year, title, journal source, the studied intervention, the number of incorporated studies, type of management, major findings, and any additional information deemed pertinent by the authors were systematically documented.

## 3. Results

### 3.1. Identification and Description of the Studies

**Figure 1:**
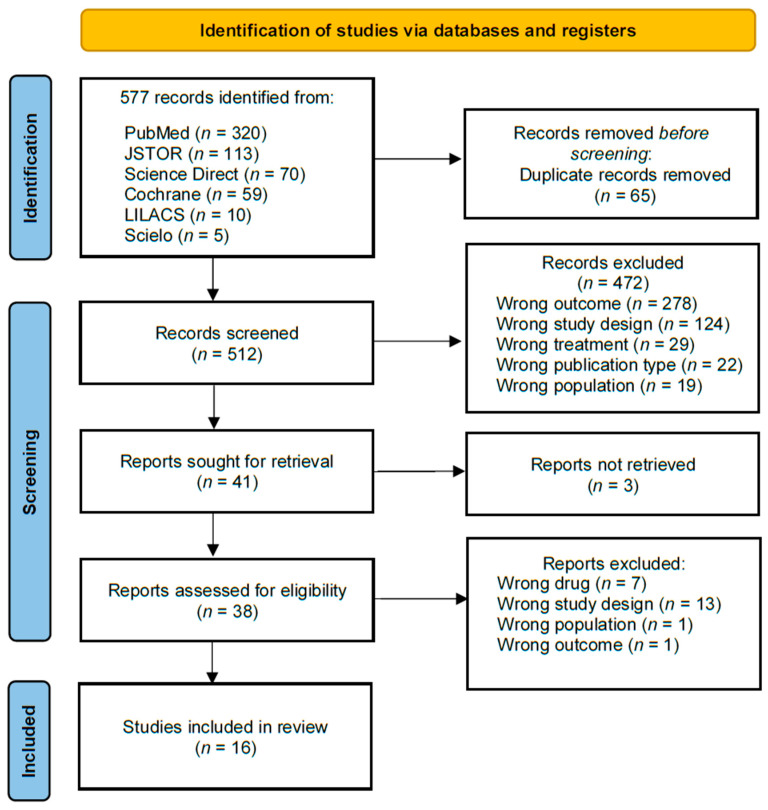
Preferred Reporting Items for Systematic Reviews and Meta-Analyses (PRISMA) flowchart.

A search conducted on 8 August 2023, identified 577 records. After deduplication, 512 studies were screened by title and abstract. Of these, forty-one were retrieved, but three were unavailable. Full-text review of 38 studies led to the exclusion of 22, resulting in 16 studies for analysis. Figure 1 depicts the PRISMA flowchart [23] for study selection and provides the reasons for study exclusion.

The included reviews investigated several approaches to the management of Crohn’s disease. Nine studies researched the use of biologic agents in Crohn’s disease, namely adalimumab [24,25,26,27], briakinumab [28,29], ustekinumab [28,29,30], and certolizumab pegol [31,32].

Regarding antimetabolites, three studies were included using methotrexate [33,34] and purine analogues azathioprine and 6-mercaptopurine [35].

Two studies that investigated integrin receptor antagonists such as vedolizumab [36,37] and natalizumab [37] were also included.

Two studies using budesonide corticosteroid were also included [38,39].

Regarding the objective, eight articles focused on evaluating interventions for the induction of remission in active Crohn’s disease [24,25,28,30,31,33,35,39]. Five articles addressed the maintenance of remission [27,29,32,34,38], while three investigated interventions for both induction and maintenance [26,36,37].

### 3.2. Quality Assessment

All studies were rated as “good” according to the previously defined parameters (Table 1). No studies presented major flaws. Fifteen of the sixteen included studies did not perform a publication bias assessment due to the low number of included studies (<10); therefore, it was not considered a flaw. A dual review for determining which studies to include and exclude was not reported in one study [26], and a dual quality appraisal for internal validity was not reported in two studies [26,35] and could not be determined in two others [24,33].

### 3.3. Effects and Adverse Events

#### 3.3.1. Induction of Remission

The characteristics of the included studies in this topic are presented in Table 2.

#### Antimetabolites

The study by McDonald et al. [33] aimed to evaluate the effectiveness and safety of methotrexate in inducing remission for active Crohn’s disease. The results of this study showed that some studies with low-dose oral methotrexate suggest no significant difference in remission rates compared to placebo or other medications. One large, high-dose intramuscular methotrexate study showed a statistically significant benefit compared to placebo. Regarding safety, methotrexate was associated with more adverse events and withdrawals due to more adverse events compared to placebo or other medications in some studies. According to the authors, the evidence is limited due to the high risk of bias as well as low to very low quality of evidence for most outcomes due to limited data and inadequate blinding.

The study of Chande et al. [35] had the objective of evaluating the effectiveness and safety of azathioprine and 6-mercaptopurine in inducing the remission of Crohn’s disease. Azathioprine/6-mercaptopurine showed a possible modest benefit over placebo in clinical remission rates (moderate quality evidence), as well as a similar trend for clinical improvement rates (moderate quality evidence). Furthermore, azathioprine showed a possible benefit over placebo in steroid-sparing (moderate quality evidence). The assessment of the safety profile showed that azathioprine/6-mercaptopurine had a possible increased risk of withdrawals due to adverse events compared to placebo (moderate quality evidence) and an increased risk of serious adverse events also compared to placebo (low-quality evidence). In addition to overall moderate quality evidence for the main outcomes, most studies showed a low risk of bias.

#### Biologic Agents

Anti-TNF agents

The aim of the study by Song et al. [26] was to evaluate the efficacy and safety of adalimumab for moderate to severe Crohn’s disease. Regarding the induction of remission, the results of this study suggest that adalimumab can produce higher rates of clinical response (a reduction in Crohn’s Disease Activity Index (CDAI) score) and remission (CDAI score < 150) compared to placebo at week 4. The clinical response rates seem to depend on dose according to some of the reviewed studies, and one study showed a trend towards mucosal healing (absence of ulceration) at week 12 in patients receiving adalimumab compared to placebo. The analysis of adverse events showed no statistically significant difference in the occurrence between the adalimumab and placebo groups. According to the quality assessment, all the studies were ranked as moderate quality due to some potential for bias.

Additionally, Abbass et al. [24] assessed the use of adalimumab for the induction of remission in participants with active moderate to severe Crohn’s disease. In their study, adalimumab showed a significant benefit over placebo in achieving clinical remission at four weeks (24% vs. 9%). Similarly, adalimumab demonstrated a significant advantage over placebo in achieving both 70-point (56% vs. 34%) and 100-point (43% vs. 24%) improvements in the Crohn’s Disease Activity Index (CDAI) score at four weeks. These results were deemed as high-certainty evidence. Adverse events were common in both groups, with a slightly lower rate observed in the adalimumab group (62% vs. 72%) with moderate-certainty evidence. Serious adverse events were rare, with a trend towards a lower rate in the adalimumab group (2% vs. 5%) with low-certainty evidence.

Yin et al. [25] also studied the efficacy and safety of adalimumab in inducing Crohn’s disease remission. At 4 weeks, adalimumab showed a significantly higher rate of clinical remission compared to placebo (27% vs. 8%). Also, adalimumab demonstrated a significantly better response compared to placebo at both 70-point (53% vs. 27%) and 100-point (43% vs. 24%) reductions. For the previous outcomes, the 160 mg/80 mg dose appeared to be the most effective, and previous exposure to anti-TNF-α therapy did not significantly influence the results. Adverse events were common in both groups, with a slightly lower rate observed in the adalimumab group (55% vs. 62%). Serious adverse events were scarce, with a trend towards a lower rate in the adalimumab group (2% vs. 4%). Clinical outcomes were considered as moderate-certainty evidence, adverse events as high-certainty evidence, and serious adverse events as low-certainty evidence.

Yamazaki et al. [31] aimed to assess the effectiveness (induction of remission) and safety of certolizumab pegol for Crohn’s disease. Regarding the results of the study, certolizumab pegol showed a statistically significant benefit over placebo in achieving clinical remission at week 8 (26.9% certolizumab pegol vs. 19.8% placebo). Similarly, certolizumab pegol demonstrated a significant advantage over placebo in achieving clinical response at week 8 (40.2% vs. 30.9%). Serious adverse events were reported in both groups, with a slightly higher rate in the certolizumab pegol group (8.7% vs. 6.2%). These three outcomes were considered as moderate-certainty evidence.

Anti-IL-12/23p40 Antibodies

MacDonald et al. [28] conducted a study that assessed the effectiveness (induction of remission) and safety of specific anti-interleukin-12/23 antibodies, such as briakinumab or ustekinumab, for Crohn’s disease. Results regarding briakinumab showed no significant difference in remission rates compared to placebo at week 6 (30% briakinumab vs. 19% placebo). Also, the subgroup analysis revealed no effect of dose on remission rates. The trials that provided these results were considered to have a low risk of bias. Ustekinumab studies showed a statistically significant difference in remission rates at week 6 compared to placebo (16% ustekinumab vs. 10% placebo). The subgroup analysis suggested that the 6 mg/kg dose was the most effective (moderate-quality evidence). Ustekinumab also showed significant improvement compared to placebo in achieving both 70-point and 100-point reductions in the CDAI score, indicating a better clinical response. The safety profile appeared similar between medication and placebo.

The study by Kawalec et al. [30] evaluated the effectiveness and safety of ustekinumab as an induction therapy for Crohn’s disease in patients who have failed anti-TNF-α therapy. The results suggest that ustekinumab was significantly better than placebo in achieving a clinical response (36.7% vs. 22.5%) at week 6. The benefit of Ustekinumab was consistent across most subgroups of patients who previously did not respond to their first course of TNF-alpha antagonists (secondary nonresponders), could not tolerate TNF-alpha antagonists due to side effects, and failed treatment with two different TNF-alpha antagonists. However, there was no significant difference in clinical response between ustekinumab and placebo in patients who initially responded well to a TNF-α antagonist (primary responders). The analysis of adverse events associated with the ustekinumab treatment for Crohn’s disease showed no significant differences compared to placebo. The quality of the studies was reported as high.

Integrin Receptor Antagonists

Chandar et al. [37] sought to explore the efficacy and safety of natalizumab and vedolizumab for the management of Crohn’s disease. Regarding the induction of remission outcomes, with a timing of up to 14 weeks, results showed that both natalizumab and vedolizumab were significantly more effective than placebo in inducing remission in patients with Crohn’s disease. Both treatment-naive patients and anti-TNF-exposed patients benefited significantly from natalizumab and vedolizumab compared to placebo. However, while there was a trend towards improved clinical response with therapy, natalizumab and vedolizumab compared to placebo, the data were less consistent across studies (high heterogeneity). Furthermore, patients treated with integrin receptor antagonist therapy did show improvement in health-related quality of life, but the change did not reach the pre-defined minimally clinically important difference. Regarding the safety profile of these anti-α4-integrins, there were no significant differences in the rates of overall serious adverse events among patients treated with natalizumab, vedolizumab, or placebo. Nevertheless, patients treated with natalizumab experienced a significantly higher rate of infusion reactions (13.5%) compared to placebo (6.9%). These reactions can be acute or resemble hypersensitivity reactions. Vedolizumab had a much lower rate of infusion reactions (2.0%) compared to natalizumab, and the difference between vedolizumab and placebo was not statistically significant. The strength of evidence across the included studies varied by outcome. Remission with natalizumab or vedolizumab in adult Crohn’s disease had high evidence, while response was moderate, and health-related quality of life showed limited evidence.

The study by Hui et al. [36] aimed to investigate whether vedolizumab is more effective and safer than a placebo in achieving and maintaining remission in people with Crohn’s disease. Considering the induction of remission results, vedolizumab was significantly more effective than placebo in inducing remission at weeks 6–10 (19.8% vs. 11.6%). This benefit was observed regardless of whether patients had previously failed TNF inhibitor medication. Vedolizumab was also more effective than placebo in inducing a clinical response (36.9% vs. 24.2%). Similar to remission, this benefit was seen in both patients who had and had not previously failed TNF inhibitors. Concerning adverse events, there was no significant difference in the rates of any adverse events (both treatment-related and unrelated) between patients treated with vedolizumab and those given placebo. The rate of serious adverse events was also similar between the vedolizumab and placebo groups. Clinical outcomes were graded as high-certainty evidence, adverse events as moderate, and serious adverse events as low.

#### Corticosteroids

The objective of the study by Rezaie et al. [39] was to evaluate the efficacy and safety profile of oral budesonide for inducing remission in the pediatric and adult populations diagnosed with active Crohn’s disease. Regarding the induction of remission in adult patients, results showed that there was not enough evidence to determine if 3 mg of budesonide is superior to a placebo for inducing remission. Budesonide at a daily dose of 9 mg was significantly more effective than placebo at inducing remission at week 2 (31% vs. 9%), week 4 (40% vs. 20%), and week 8 (47% vs. 22%). Budesonide at a daily dose of 15 mg was also more effective than placebo at week 2 (24% vs. 8%), week 4 (36% vs. 15%), and week 8 (39% vs. 17%).

The quality of evidence for these outcomes was low for 3 mg and moderate for 9 mg and 15 mg due to sparse data. Changes in the Crohn’s Disease Activity Index scores could not be calculated due to a lack of information in the studies. The quality of life outcomes measured using the inflammatory bowel disease questionnaire showed no statistically significant difference in scores between patients receiving 9 mg of budesonide and those on placebo. Concerning the safety profile of budesonide, 3 mg and 15 mg did not show significant differences in abnormal hormone tests or steroid-related side effects in comparison to placebo. Meanwhile, despite similar steroid-related side effects compared to placebo, the use of 9 mg of budesonide was linked to a higher risk of impaired adrenal function (more than double the risk of displaying an abnormal ACTH test).

#### 3.3.2. Maintenance of Remission

The characteristics of the included studies in this topic are presented in Table 3.

#### Antimetabolites

The study by Patel et al. [34] examines the efficacy and safety of methotrexate for maintaining remission in patients with quiescent Crohn’s disease. Their results suggest that oral methotrexate (12.5 mg/week) has no significant difference compared to placebo in maintaining remission at 36 weeks (90% methotrexate vs. 67% placebo). The quality of the evidence is low due to very limited data. On the other hand, intramuscular methotrexate was shown to be superior to placebo for maintaining remission at 40 weeks (65% vs. 39%). The quality of the evidence is moderate also due to sparse data. According to the authors, methotrexate exhibited a favourable safety profile. Adverse events were generally mild and transient, resolving upon the discontinuation of treatment or with folic acid supplementation.

#### Biologic Agents

Anti-TNF Agents

The study by Song et al. [26] examines the effectiveness of adalimumab in maintaining response and remission in patients with moderate to severe Crohn’s Disease who initially responded well to adalimumab treatment. Compared to placebo, adalimumab significantly increased the chances of maintaining remission at week 26 (40%/47% [40 mg every other week/every week] vs. 17% [placebo]), at week 52 (38.1% vs. 9.1%), and at week 56 (79%/83% [every other week/every week] vs. 44%). Regarding fistula treatment, the effectiveness of adalimumab in achieving complete fistula closure was considered inconclusive due to very limited available data and mixed results. Moreover, compared to placebo, adalimumab significantly increased the chances of short-term remission in patients who previously received the anti-TNF agent infliximab. Also, limited data suggests adalimumab might also be beneficial for long-term remission in these patients. It is also suggested that adalimumab is effective for patients with prior infliximab exposure. However, its effectiveness might be lower compared to patients who are naive to infliximab treatment. Concerning adverse and severe adverse events, the authors’ results suggest no difference between adalimumab and placebo. As reported previously, according to the quality assessment, all the studies were ranked as moderate quality due to some potential for bias.

Townsend et al. [27] conducted a study to evaluate the efficacy and safety of adalimumab for the maintenance of remission in people with quiescent Crohn’s disease. Their results suggest that adalimumab was significantly more effective than placebo in maintaining remission (41% vs. 14%) at 52 to 56 weeks (high-certainty evidence). Also, compared to placebo, adalimumab showed a potential early benefit (24–26 weeks) in maintaining remission (48% vs. 21%, moderate-certainty evidence). In maintaining response, adalimumab was also significantly more effective than placebo (46% vs. 19%). Adalimumab showed limited benefit in maintaining endoscopic remission (28% vs. 3%, moderate-certainty evidence), response (24% vs. 0%), or histological remission (24% vs. 5%) at 52 weeks compared to placebo. No significant difference was detected for adverse events (87% adalimumab vs. 85% placebo, moderate-certainty evidence).

Okabayashi et al. [32] assessed the use of certolizumab pegol for the maintenance of medically induced remission in patients with Crohn’s disease. Only one study was included in this review. Regarding clinical remission maintenance, certolizumab pegol was superior to placebo at week 26 (47.7% vs. 28.3%). The same occurred concerning the maintenance of clinical response (62.5% vs. 35.8%). Outcomes were considered as moderate-certainty evidence. Health-related quality of life was also assessed, and the results showed no differences between certolizumab pegol and placebo. Both adverse events and serious adverse events were similar between groups (adverse events: 64.8% certolizumab pegol vs. 67.5% placebo and serious adverse events: 5.6% certolizumab pegol vs. 6.6% placebo, low certainty evidence).

Anti-IL-12/23p40 Antibodies

Davies et al. [29] assessed the efficacy and safety of two anti-IL-12/23p40 antibodies for the maintenance of remission in Crohn’s disease. Regarding ustekinumab, participants showed superior results in maintaining remission compared to placebo at week 22 (42% vs. 27%). This trend continued at 44 weeks, with ustekinumab recipients having a higher chance of maintaining remission compared to placebo (51% vs. 36%). Moreover, ustekinumab showed superiority in maintaining clinical response compared to placebo at 22 weeks (69% vs. 42%). Also, at week 44, ustekinumab showed better results compared to placebo (59% vs. 44%). These outcomes were graded as moderate-certainty evidence due to sparse data. As for the occurrence of adverse events, there seems to be no significant difference between ustekinumab and placebo (80% ustekinumab vs. 84% placebo, high-certainty evidence). Additionally, ustekinumab and placebo showed similar levels of serious adverse events (11% ustekinumab vs. 16% placebo, moderate-certainty evidence).

Concerning briakinumab, one small study was included and showed that, at 24 weeks, the maintenance remission levels were similar between briakinumab and placebo (49% briakinumab vs. 39% placebo). Also, the maintenance of clinical response showed the same trend (66% briakinumab vs. 47% placebo). Both outcomes were considered as low-certainty evidence. There was no significant difference in remission and response rates between the different briakinumab dosage groups (200 mg, 400 mg, and 700 mg). Adverse events were similar (66% briakinumab vs. 64% placebo), and serious adverse events were fewer in the briakinumab group (2% vs. 7%). Both outcomes were graded as low-certainty evidence due to sparse data.

Integrin Receptor Antagonists

As mentioned before, Chandar et al. [37] sought to explore the efficacy and safety of natalizumab and vedolizumab for the management of Crohn’s disease. Regarding maintenance therapy, both anti-α4-integrins (one study for each) seem effective in maintaining remission compared to placebo. At week 36, natalizumab showed superiority in comparison with placebo (61% vs. 28%), with benefits up to week 60. At week 52, vedolizumab also achieved superior results compared to placebo when taken every 4 weeks or every 8 weeks (36.5% every 4 weeks/39% every 8 weeks vs. 21.6%). Clinical maintenance response was also evaluated, and vedolizumab showed superior results compared to placebo (48% every 4 weeks/46% every 8 weeks vs. 32%). Concerning adverse events, there were no differences between natalizumab and placebo (serious adverse events: 8.4% natalizumab vs. 9.8% placebo, infusion reaction: 9.3% natalizumab vs. 9.3% placebo, infections: 61.7% natalizumab vs. 55.6% placebo, and serious infections: 2.8% natalizumab vs. 2.3% placebo). Also, no differences were observed between vedolizumab and placebo (serious adverse events: 24.4% vedolizumab vs. 15.3% placebo, infusion reaction: 4.1% vedolizumab vs. 4.7% placebo, infections: 44.1% vedolizumab vs. 40.2% placebo, and serious infections: 5.5% vedolizumab vs. 3% placebo).

Also mentioned before, Hui et al. [36] focused on studying the efficacy of vedolizumab in achieving and maintaining remission in people with Crohn’s disease. In the included studies, participants received either vedolizumab or placebo after initially responding well to vedolizumab treatment. According to the results, vedolizumab was significantly more effective than placebo in maintaining remission at one year (42.5% vs. 27.1%, high-certainty evidence). The benefit of vedolizumab was consistent regardless of whether patients had previously failed anti-TNF medications (not previously failed: 49.1% vedolizumab vs. 34.8% placebo and previously failed: 36.8% vedolizumab vs. 18.8% placebo). Adverse events were high but not statistically different between vedolizumab and placebo (80% vedolizumab vs. 80.3% placebo). Additionally, serious side effects occurred in a similar proportion of patients treated with vedolizumab and placebo (13.1% vedolizumab vs. 13.7% placebo). While results suggest no significant difference in side effects between the medication and placebo, the certainty of this finding is low to moderate. This is because the studies have a moderate chance of being inaccurate due to wide confidence intervals around the effect size estimate.

#### Corticosteroids

Kuenzig et al. [38] conducted a study in which the primary objective was to assess the efficacy and safety of budesonide therapy for the maintenance of remission in Crohn’s disease. Budesonide at 6 mg did not show a statistically significant difference in maintenance when compared to placebo at 3 months (64% budesonide vs. 52% placebo, low-certainty evidence due to moderate heterogeneity and sparse data). Similarly, budesonide at 6 mg was not superior to placebo at 6 months (61% budesonide vs. 52% placebo, moderate-certainty evidence) and 12 months (55% budesonide vs. 48% placebo, moderate-certainty evidence). Regarding budesonide at 3 mg, the corticosteroid was superior to placebo at 3 months (57% budesonide vs. 44% placebo, moderate-certainty evidence). However, there was no statistically significant difference at 6 months (49% budesonide vs. 44% placebo, moderate-certainty evidence) and 12 months (42% budesonide vs. 40% placebo, moderate-certainty evidence). No differences were found regarding adverse events for budesonide at 6 mg or 3 mg in comparison to placebo; however, both budesonide doses were associated with a significantly higher rate of abnormal ACTH stimulation tests compared to placebo.

## 4. Discussion

In our study, we evaluate and synthesize the existing body of systematic reviews and meta-analyses pertaining to the management strategies employed for Crohn’s disease.

In Crohn’s disease, remission can be defined as the absence of biological signs associated with the risk of short-term relapse and mid/long-term relapse [40]. Inducing remission, therefore, aims to achieve a state where patients experience minimal to no symptoms of the disease.

On the other hand, maintenance therapy aims to extend remission periods and potentially improve overall quality of life by managing the disease when it is in a low-activity or inactive state [41].

Based on current evidence, several approaches can be used to achieve these goals.

Antimetabolites constitute a significant group of anticancer agents that structurally mimic natural substrates but differ enough to disrupt their metabolism [42]. In this review, this group includes folic acid antagonists like methotrexate and purine antimetabolites such as 6-mercaptopurine and azathioprine. Antimetabolites interfere with nucleic acid synthesis either by disrupting the production of key nucleotide metabolites or by substituting these natural metabolites [42].

In autoimmune diseases, methotrexate is chosen as a drug of choice due to several mechanisms. It inhibits the enzyme AICAR transformylase, disrupting adenosine and guanine metabolism and leading to adenosine accumulation. The anti-inflammatory effects of adenosine result in the suppression of T-cell activation and the down regulation of B cells. Additionally, methotrexate increases the sensitivity of activated CD-95 T cells and represses methyltransferase activity, which inhibits the binding of beta-1 interleukin to its cell surface receptor [43].

In our study, we found that low-dose oral methotrexate does not seem to have more efficacy than a placebo. However, the application of a high dose of intramuscular methotrexate seems to be positive in inducing remission. In clinical practice, intramuscular administration means a frequent visit to the clinic, implicating higher costs and reduced quality of life for patients [33]. Concerning the maintenance of remission, using oral methotrexate did not show efficacy at 36 weeks, while intramuscular methotrexate was shown to be superior to placebo for maintaining remission at 40 weeks.

In addition, this administration resulted in a higher incidence of adverse events. Common adverse events include nausea, vomiting, abdominal pain, diarrhea, skin rash, and headache. Similar to most cytotoxic anticancer agents, antimetabolites are toxic to normal cells, particularly those in the bone marrow and gastrointestinal tract [42]. After initiating methotrexate therapy, follow-up tests should include monitoring complete blood count, renal function, and liver function tests. These tests are recommended weekly for the first 4 weeks and then at least every two months thereafter [34]. Moreover, interactions with other medications are common. NSAIDs, salicylates, TMP, penicillin, warfarin, valproate, proton pump inhibitors, cyclosporin, and cisplatin increase the risk of methotrexate toxicity in the blood, and aminoglycosides, neomycin, and probenecid reduce the absorption of methotrexate [44,45].

Thiopurine drugs like azathioprine undergo complex metabolism. Azathioprine is converted into 6-mercaptopurine and methyl-nitro-thioimidazole by glutathione-S-transferase. Within cells, 6- mercaptopurine faces three competing enzyme pathways: xanthine oxidase produces inactive metabolites; allopurinol inhibits xanthine oxidoreductase, leading to increased production of alternative metabolites; and hypoxanthine guanine phosphoribosyltransferase produces active metabolites [46].

In this review, azathioprine and 6-mercaptopurine appear to offer a slight benefit over placebo in achieving remission or clinical improvement for active Crohn’s disease, allowing patients to reduce steroid use. However, it is important to note that side effects are more common in patients taking these medications compared to those on placebo. Common adverse events included allergic reactions, leukopenia, pancreatitis, and nausea, according to Chande et al. [35]. The toxicity of these drugs is linked to the relative concentrations of metabolites (thiopurine methyl-transferase influences metabolite concentration), and genetic variations in metabolizing enzymes contribute to significant differences in individual drug responses and susceptibility to toxicity [46]. Also, there is a high number of drug interactions [47]. No studies were included regarding the maintenance of remission.

According to Crohn’s & Colitis UK [48], biologic agent medications are a class of drugs that specifically block key molecules involved in the inflammatory response of Crohn’s disease.

Adalimumab and certolizumab pegol are anti-TNF agents that neutralize tumour necrosis factor-α (TNF-α), consequently inhibiting the inflammatory response [49,50]. These drugs may achieve this by affecting multiple pathways, including potentially blocking LPS-induced IL-1β release from monocytes [50].

Regarding clinical efficacy, the studies reported in this review showed that adalimumab is superior to placebo in inducing remission at 4 weeks. Additionally, adalimumab seems to be effective in maintaining remission at weeks 26, 52, and 56.

Certolizumab pegol also showed positive results at 8 weeks; however, rates of remissions were low. For the maintenance of remission and the maintenance of response, certolizumab pegol was superior to placebo at week 26. Health-related quality of life did not show improvements. Only one meta-analysis (assessing four studies) evaluated the efficacy of certolizumab pegol. Therefore, more studies are needed to confirm its efficacy.

Overall, both treatments seemed to be safe in comparison to placebo. However, the administration process (injection) for these medications involves regular visits to a healthcare facility, or proper training is necessary for self-administration.

Other biologic agents were reported in our results, namely anti-IL-12/23 antibodies briakinumab and ustekinumab. Both monoclonal antibodies were developed to target the immune system and treat autoimmune diseases. The main difference between both medications is that briakinumab targets two specific proteins, interleukin-12 (IL-12) and interleukin-23 (IL-23), while ustekinumab specifically targets interleukin-12 and interleukin-23 (IL-12/23) p40 subunit. Targeting these proteins will inhibit Th17 cell development [51], which is related to abnormal immune responses and autoimmune diseases, including inflammatory bowel disease [52,53]. According to our results, briakinumab did not show a superior remission induction effect compared to placebo at week 6. Also, the maintenance of remission and clinical responses were similar to placebo. On the other hand, ustekinumab showed positive results in remission and clinical response at week 6, displaying a safe profile. Limited studies concerning maintenance efficacy showed that ustekinumab is superior to placebo in maintaining remission and clinical response at weeks 22 and 44.

Anti-α4-integrins such as natalizumab and vedolizumab are also biologic agents reported in our results.

Integrins are a family of cell adhesion molecules that play a critical role in mediating leukocyte trafficking and retention within tissues. Their function in directing immune cell migration to specific locations suggests a potential involvement in the pathogenesis of inflammatory bowel disease [54].

Supporting this hypothesis, a prior genome-wide association study [55] identified associations between inflammatory bowel disease susceptibility and increased immune activation of specific integrin genes (ITGA4, ITGB8, ITGAL, and ICAM1). Furthermore, these loci were not only associated with inflammatory bowel disease but also with a heightened risk of disease development in the studied population.

Further strengthening the link between integrins and inflammatory bowel disease, studies have demonstrated an upregulated expression of gut-tropic integrins and associated cell adhesion molecules within inflamed intestinal tissues of inflammatory bowel disease patients. This localized upregulation suggests a potential role for these factors in the ongoing immune cell recruitment and adhesion characteristic of the inflammatory response observed in inflammatory bowel disease [54].

Natalizumab is a humanized immunoglobulin G4 (IgG4) monoclonal antibody specifically designed to target the α4 subunit of the integrin heterodimer. This targeted action effectively inhibits both α4β1 and α4β7 integrin subtypes. Consequently, natalizumab disrupts leukocyte adhesion and migration from the vasculature into the surrounding tissues [56]. On the other hand, vedolizumab specifically targets the α4β7 integrin, blocking the interaction between α4β7 and MAdCAM-1 (mucosal addressin cell adhesion molecule 1). This molecule is primarily expressed on the endothelium (lining) of blood vessels in the gut. By blocking this interaction, vedolizumab prevents leukocyte adhesion specifically to the intestinal endothelium, thereby limiting their migration into the gut tissue. Vedolizumab does not affect the interaction of α4β1 integrin with VCAM-1, which is found on various endothelial cells throughout the body [54,57].

Our findings indicate that both natalizumab and vedolizumab achieved superior clinical remission rates compared to placebo. However, no significant differences in quality of life were observed between the medication and placebo. A very limited number of maintenance studies also show that these medications are effective in maintaining remission and response.

Regarding safety, both drugs demonstrated an overall favourable profile. However, natalizumab was associated with a higher risk of experiencing adverse infusion reactions compared to vedolizumab and placebo in the induction of the remission phase.

Finally, budesonide is a medication of the corticosteroid class. This therapeutic option stands out in inflammatory bowel disease treatment due to its targeted action within the inflamed intestine and minimal systemic exposure [58]. The details of the anti-inflammatory mechanism involve a three-step cascade: (1) Upon entering the cell, a glucocorticoid molecule binds to a specific receptor located in the cytoplasm. This binding triggers the receptor’s activation; (2) the activated glucocorticoid–receptor complex undergoes a transformation, allowing it to migrate from the cytoplasm into the cell’s nucleus; and (3) once inside the nucleus, the complex binds to specific regions of the DNA, regulating the expression of certain genes. One key gene product induced by this process is a group of proteins called lipocortins. These newly synthesized lipocortins play a crucial role by inhibiting phospholipase A2, a critical enzyme in the arachidonic acid cascade. The arachidonic acid cascade is a prominent pathway involved in inflammation, and inhibiting phospholipase A2 disrupts the production of inflammatory mediators, ultimately leading to a dampening of the inflammatory response [59]. The results of this review indicate that at a 3 mg dose, budesonide effects are comparable to those of a placebo for remission induction but superior for maintenance of remission at 3 months (not at 6 or 12 months). Both 9 mg and 15 mg demonstrated significantly improved remission rates compared to placebo, while 6 mg was effective for maintenance at 12 months (not at 3 months). However, it is important to note that the 9 mg induction dose and the 3 mg and 6 mg maintenance doses are associated with an increased risk of adrenal gland suppression. This potential for adrenal suppression necessitates close monitoring of patients receiving this medication. This is particularly important because adrenal insufficiency, a life-threatening condition characterized by insufficient cortisol production by the adrenal glands, can arise from the dysfunction of the hypothalamic–pituitary–adrenal (HPA) axis [60,61].

The review of the included studies yielded generally positive results for the therapeutic options investigated. However, several key limitations warrant consideration.

A notable limitation is the relatively small number of studies available for most of the analyzed medications. This highlights the need for further research to strengthen the evidence base for these interventions. Also, overall efficacy across the therapeutic options appears modest, with most not achieving a response rate exceeding 40 to 50% of patients, whether for induction or maintenance therapy.

Furthermore, adverse events constitute a significant factor to be weighed when assessing therapeutic options. The included studies data should be interpreted cautiously as patients in clinical trials often do not represent patients seen in clinical practice, sample sizes are not large enough, or the follow-up may not be sufficiently long to observe some serious adverse events, according to Song et al. [26]. Therefore, further longer-duration studies (12+ months) and a larger number of participants are still required to assess the safety profile of these medications.

Future research directions could focus on larger, well-designed clinical trials to confirm the efficacy of the most promising options. In addition, the evaluation of novel therapeutic strategies with the potential to achieve higher response rates is needed.

## 5. Conclusions

Current meta-analyses suggest that biologic therapies are considered the most effective treatment options for both the induction and maintenance of remission in Crohn’s Disease. Among these, adalimumab, ustekinumab, and vedolizumab provide the strongest evidence for efficacy in achieving and sustaining clinical remission. While these options show promise, limitations highlight the need for further research. Overall efficacy across treatments remains modest, with most not exceeding a 40–50% response rate. Future research should focus on larger studies and the development of novel therapeutic strategies with the potential for higher efficacy and fewer side effects.

## Figures and Tables

**Table 1:** Quality assessment of the included studies according to the Quality Assessment Tool for Systematic Reviews and Meta-Analyses [22].

	Q1	Q2	Q3	Q4	Q5	Q6	Q7	Q8
McDonald et al. [33]	Yes	Yes	Yes	Yes	CD	Yes	NA	Yes
Chande et al. [35]	Yes	Yes	Yes	Yes	NR	Yes	Yes	Yes
Rezaie et al. [39]	Yes	Yes	Yes	Yes	Yes	Yes	NA	Yes
MacDonald et al. [28]	Yes	Yes	Yes	Yes	Yes	Yes	NA	Yes
Kawalec et al. [30]	Yes	Yes	Yes	Yes	Yes	Yes	NA	Yes
Yamazaki et al. [31]	Yes	Yes	Yes	Yes	Yes	Yes	NA	Yes
Abbass et al. [24]	Yes	Yes	Yes	Yes	CD	Yes	NA	Yes
Yin et al. [25]	Yes	Yes	Yes	Yes	Yes	Yes	NA	Yes
Song et al. [26]	Yes	Yes	Yes	NR	NR	Yes	NA	Yes
Chandar et al. [37]	Yes	Yes	Yes	Yes	Yes	Yes	NA	Yes
Hui et al. [36]	Yes	Yes	Yes	Yes	Yes	Yes	NA	Yes
Kuenzig et al. [38]	Yes	Yes	Yes	Yes	Yes	Yes	NA	Yes
Patel et al. [34]	Yes	Yes	Yes	Yes	Yes	Yes	NA	Yes
Davies et al. [29]	Yes	Yes	Yes	Yes	Yes	Yes	NA	Yes
Townsend et al. [27]	Yes	Yes	Yes	Yes	Yes	Yes	NA	Yes
Okabayashi et al. [32]	Yes	Yes	Yes	Yes	Yes	Yes	NA	Yes

Q1—focused research question; Q2—eligibility criteria; Q3—literature search; Q4—dual review for inclusion/exclusion; Q5—quality appraisal; Q6—studies’ description; Q7—publication bias analysis; Q8—heterogeneity analysis; NR—not reported; CD—cannot determine; NA—not applicable.

**Table 2:** Included induction of remission studies’ characteristics.

	Intervention	Category	Nu. of RCTs	Nu. Placebo-Controlled RCTs
McDonald et al. [33]	Methotrexate	Antimetabolites	7 (495 patients)	3
Chande et al. [35]	Purine analogues (Azathioprine, 6-mercaptopurine)	Antimetabolites	13 (1211 patients)	9
Song et al. [26]	Anti-TNF agent Adalimumab	Biologic agents	6 (1676 patients)	4
Abbass et al. [24]	Anti-TNF agent Adalimumab	Biologic agents	3 (714 patients)	3
Yin et al. [25]	Anti-TNF agent Adalimumab	Biologic agents	4 (919 patients)	4
Yamazaki et al. [31]	Anti-TNF agent Certolizumab pegol	Biologic agents	4 (1485 patients)	4
MacDonald et al. [28]	Anti-IL-12/23p40 antibodies (Briakinumab or Ustekinumab)	Biologic agents	6 (2324 patients)	6
Kawalec et al. [30]	Anti-IL-12/23p40 antibodies Ustekinumab	Biologic agents	3 (not reported)	2
Chandar et al. [37]	Anti-α4-integrins (Natalizumab and Vedolizumab)	Biologic agents	8 (2672 patients)	8
Hui et al. [36]	Anti-α4-integrins Vedolizumab	Biologic agents	5 (2020 patients)	4
Rezaie et al. [39]	Budesonide	Corticosteroids	14 (1805 patients)	3

RCTs: randomized controlled trials.

**Table 3:** Included maintenance of remission studies’ characteristics.

	Intervention	Category	Nu. of RCTs	Nu. Placebo-Controlled RCTs
Patel et al. [34]	Methotrexate	Antimetabolites	5 (333 patients)	2
Song et al. [26]	Anti-TNF agent (Adalimumab)	Biologic agents	6 (1676 patients)	6
Townsend et al. [27]	Anti-TNF agent (Adalimumab)	Biologic agents	6 (1158 patients)	4
Okabayashi et al. [32]	Anti-TNF agent (Certolizumab pegol)	Biologic agents	1 (428 patients)	1
Davies et al. [29]	Anti-IL-12/23p40 antibodies (Briakinumab or Ustekinumab)	Biologic agents	3 (646 patients)	3
Chandar et al. [37]	Anti-α4-integrins (Natalizumab and Vedolizumab)	Biologic agents	8 (2672 patients)	2
Hui et al. [36]	Anti-α4-integrins (Vedolizumab)	Biologic agents	5 (2020 patients)	3
Kuenzig et al. [38]	Budesonide	Corticosteroids	12 (1273 patients)	8

RCTs: randomized controlled trials.

## Data Availability

The original contributions presented in this study are included in the article. Further inquiries can be directed to the corresponding author.
